# Effects of Dairy Products Consumption on Health: Benefits and Beliefs*—*A Commentary from the Belgian Bone Club and the European Society for Clinical and Economic Aspects of Osteoporosis, Osteoarthritis and Musculoskeletal Diseases

**DOI:** 10.1007/s00223-015-0062-x

**Published:** 2015-10-07

**Authors:** Serge Rozenberg, Jean-Jacques Body, Olivier Bruyère, Pierre Bergmann, Maria Luisa Brandi, Cyrus Cooper, Jean-Pierre Devogelaer, Evelien Gielen, Stefan Goemaere, Jean-Marc Kaufman, René Rizzoli, Jean-Yves Reginster

**Affiliations:** Department of Public Health, Epidemiology and Health Economics, University of Liège, Liège, Belgium; Department of Gynaecology-Obstetrics, Université Libre de Bruxelles, Brussels, Belgium; Department of Medicine, CHU Brugmann, Université Libre de Bruxelles, Brussels, Belgium; Department of Radioisotopes, CHU Brugmann, Université Libre de Bruxelles, Brussels, Belgium; Metabolic Bone Unit, Department of Internal Medicine, University of Florence, Florence, Italy; MRC Lifecourse Epidemiology Unit, University of Southampton, Southampton, UK; University of Oxford, Oxford, UK; Department of Rheumatology, Saint Luc University Hospital, Université Catholique de Louvain, Louvain-la-Neuve, Belgium; Gerontology and Geriatrics Section, Department of Experimental Medicine, Katholiek Universiteit Leuven, Leuven, Belgium; Department of Rheumatology and Endocrinology, State University of Ghent, Ghent, Belgium; Department of Endocrinology, State University of Ghent, Ghent, Belgium; Division of Bones Diseases, Geneva University Hospitals and Faculty of Medicine, Geneva, Switzerland

**Keywords:** Dairy products, Cardiovascular disease, Osteoporosis, Arthritis, Weight management, Lactose intolerance

## Abstract

Dairy products provide a package of essential nutrients that is difficult to obtain in low-dairy or dairy-free diets, and for many people it is not possible to achieve recommended daily calcium intakes with a dairy-free diet. Despite the established benefits for bone health, some people avoid dairy in their diet due to beliefs that dairy may be detrimental to health, especially in those with weight management issues, lactose intolerance, osteoarthritis, rheumatoid arthritis, or trying to avoid cardiovascular disease. This review provides information for health professionals to enable them to help their patients make informed decisions about consuming dairy products as part of a balanced diet. There may be a weak association between dairy consumption and a possible small weight reduction, with decreases in fat mass and waist circumference and increases in lean body mass. Lactose intolerant individuals may not need to completely eliminate dairy products from their diet, as both yogurt and hard cheese are well tolerated. Among people with arthritis, there is no evidence for a benefit to avoid dairy consumption. Dairy products do not increase the risk of cardiovascular disease, particularly if low fat. Intake of up to three servings of dairy products per day appears to be safe and may confer a favourable benefit with regard to bone health.

## Introduction

Dairy products have been an important part of the human diet for some 8000 years and are part of the official nutritional recommendations in many countries worldwide. They provide a package of key nutrients that are difficult to obtain in diets with limited or no dairy products, such as vegan or dairy restrictive diets. Indeed, dairy products are rich in calcium, protein, potassium and phosphorus. They contribute around 52–65 % of the dietary reference intake (DRI) of calcium and 20–28 % of the protein requirement, depending on the age of the consumer [1–5]. The contribution of dairy products to providing recommended calcium intakes has largely driven the dietary recommendations for dairy intake in most guidelines. Up to two-thirds of the population’s calcium intake in Western countries is supplied by dairy products [6, 7], while at the same time dairy foods represent only 9–12 % of the total energy consumption [8].

Much research is still ongoing into the health effects of dairy product consumption. This article examines some clinical evidence and provides information for health professionals to enable them to help their patients make informed decisions about consuming dairy products as part of a balanced diet. In particular, the effect of dairy products on bone health, obesity, arthritis and cardiovascular disease are briefly discussed.

## Methods

This commentary is based on a narrative literature review. It focusses on the most robust available evidence where possible, for example meta-analyses and prospective studies, with the most recent publications consulted. Relevant articles were identified through a systematic search, from 1966 to 2013 in MEDLINE with the keywords «dairy products», «bone» and «muscle». Only articles published in English were considered. Following this extensive search of the literature, a critical appraisal was obtained through three face-to-face consensus experts meetings, held during the second part of the year 2013 and the first part of the year 2014. For the purposes of this article, dairy products refer to animal milks and derived products, excluding butter and vegetable-derived products like soy or almond ‘milks’.

## Dairy Products as a Source of Key Nutrients

Worldwide, many people fail to achieve an adequate dietary calcium intake. The adequacy of dietary calcium consumption varies geographically and reflects milk consumption. Intakes of calcium are generally low across Europe [9]; judged against the World Health Organization/Food and Agriculture Organization of the United Nations (WHO/FAO) adult recommend nutrient intake (RNI) of 1000 mg/d, mean calcium intakes of 16 European countries were 687–1171 mg/day in men and 508–1047 mg/d in women [9]. Other nations fall far short of RNIs; for example, in Brazil 99 % of adults (19–60 years) consume inadequate levels of calcium [10].

To meet the daily dietary calcium requirement, dairy products, green vegetables and mineral waters are important, easily available, sources of calcium. Indeed, dairy products represent good dietary sources of calcium due to their high calcium and nutrient contents, high absorptive rate, availability and relatively low cost, which makes the regular consumption of dairy products feasible. They provide more calcium, protein, magnesium, potassium, zinc and phosphorus per calorie than any other typical food found in the adult diet [11, 12]. Many dietary recommendations include the consumption of 3 servings of dairy products per day (for example, 1 glass of milk, 1 portion of cheese, 1 yogurt)—an amount that provides most of the DRI of calcium for the general population [13]. For example, 250 mg of calcium may be obtained from a 200 ml glass of milk, a 125 g serving of yogurt or 35 g of hard cheese (Table 1) [14].

**Table 1:** Essential nutrient content per 100 g of selected dairy foods

Dairy food (food code)	Calcium (mg)	Potassium (mg)	Phosphorus (mg)	Magnesium (mg)	Zinc (mg)	Protein (g)
Milk, full-fat 3.7 % (01078)	119	151	93	13	0.38	3.3
Milk, skimmed (01151)	122	156	101	11	0.42	3.4
Yogurt, plain low-fat (01117)^a^	183	234	144	17	0.89	5.3
Yogurt, fruit low-fat (01122)^a^	169	216	133	16	0.82	4.9
Cheddar cheese (01009)	721	98	512	28	3.11	24.9
Cottage cheese, non-fat (01014)	86	137	190	11	0.47	10.3
Ice cream, vanilla (19095)	128	199	105	14	0.69	3.5

*Source* Compiled from U.S. Department of Agriculture, Agricultural Research Service. 2013. USDA National Nutrient Database for Standard Reference, Release 26. Available from: Nutrient Data Laboratory Home Page: http://www.ars.usda.gov/ba/bhnrc/ndl [14]

^a^A higher content of calcium and protein may be found in American yogurts compared with those found in Europe, as milk powder is added to thicken the consistency. In Europe, the calcium and protein contents of yogurts are similar to those of milk

There are non-dairy sources of calcium, such as mineral water, kale and dark greens, dried beans and legumes, but it is difficult to meet daily requirements with these. A variety of calcium-fortified foods, such as orange juice and soy juice, are now available and provide the same amount of calcium as a single serving of dairy; the amounts have been evaluated at 1.1 servings of fortified soy beverage, 0.6 servings of fortified orange juice, 1.2 servings of bony fish or 2.2 servings of leafy greens [15]. However, these foods do not provide the equivalent profile of other nutrients and the amounts needed are unrealistic in some cases [16]. Using data from the National Health and Nutrition Examination Survey (NHANES), it has been determined that it is impossible to meet calcium recommendations while meeting other nutrient recommendations with a dairy-free diet within the current US dietary pattern [17]. The nutrients most at risk if dairy products are excluded are calcium, potassium and magnesium. For women of 19–50 years of age who do not consume dairy products, only 44 % of calcium and 57 % of magnesium and potassium recommendations are met [18].

Under normal dietary conditions, about 30–40 % of the calcium contained in milk and cheese is absorbed in the gut either through vitamin D-dependent transport across the duodenum, facilitated diffusion or under the influence of lactose in the distal small intestine via the paracellular route [12]. In contrast, only 28–36 % of the calcium is absorbed from fortified cereals, soy (if not dephytized) and rice juice [19]. Calcium is found in green leafy vegetables in reasonable quantity (Table 2**)** [20]; however, a high proportion of the calcium is made insoluble by the presence of fibres, phytic acid and oxalic acid, which reduce the bioavailability of calcium. For example, cooked spinach contains 115 mg of calcium per serving but only 5 % of spinach calcium is absorbed, as spinach contains a high proportion of oxalates and phytates, which bind calcium and form insoluble salt compounds [20]. Thus, while consumption of 1 cup of milk per day may be considered feasible to provide 100 mg of absorbed calcium, the consumption of 16 servings of spinach per day to provide the same amount of bioavailable calcium may be considered unpalatable (Table 2).

**Table 2:** Comparison of the amount of absorbable calcium in calcium-rich foods

Food	Standard serving size^a^ (g)	Calcium content/serving (mg)	Calcium absorbed/serving (mg)	Servings needed to equal 240 ml milk
Milk	240	300	96	1.0
Yogurt	240	300	96	1.0
Cheddar cheese	42	303	97	1.0
Tofu with calcium	126	258	80	1.2
Bok choy	85	79	43	2.3
Kale	85	61	30	3.2
Broccoli	71	35	21	4.5
Spinach	85	115	6	16.3
Red beans	172	41	10	9.7
White beans	110	113	25	3.9
Pinto beans	86	45	12	8.1
Rhubarb	120	174	10	9.5

*Source* Adapted from Weaver 1999 [20]

^a^1 serving = 240 ml milk; 42 g (1.5 oz) cheese; 85 g green leafy vegetables

Dairy foods also contain protein, with a 200 ml glass of milk also providing around 6–7 g of mostly casein and whey proteins [21]. The casein phosphopeptides (CPP) and lactose in dairy foods can facilitate intestinal calcium absorption [6, 22]. For example, the enzymatic hydrolysis of casein protein leads to the formation of CPP. These molecular species have been shown to bind calcium and therefore protect it against precipitation with anions such as phosphates in the small intestine. The net result is an increase of passive calcium absorption in the ileum [23].

Phosphorus (P) ions are also present in milk, generally combined with proteins and peptides. At the kidney level, increased inorganic phosphate intake leads to decreased urinary calcium and increased calcium retention. During growth and adulthood, the administration of calcium and inorganic phosphate in a ratio close to that found in dairy products leads to positive effects on bone health [24]. Calcium and inorganic phosphate interact at both the intestinal and renal levels and impact on bone maintenance and osteoporosis management. Interactions between calcium, inorganic phosphate, protein and vitamin D reduce bone resorption, attenuating age-related bone loss [24].

### Dairy Foods or Calcium Supplements?

Increased awareness about the impact of nutrition on health and the availability of dietary supplements in shops has made them popular choices, with 43 % of Americans using calcium supplements [25]. The absorption of calcium from commonly used supplements, such as calcium carbonate and calcium citrate, is around 30–40 % and is optimized by concurrent food intake and by taking divided doses with protein-containing meals throughout the day [6]. However, calcium supplements can interact with several medications: the absorption of calcium from calcium carbonate is reduced by proton pump inhibitors, while calcium supplements may interact with antibiotics, thiazide diuretics, digoxin and phenytoin. Furthermore, calcium supplements do not contain the additional nutrients—including protein, potassium and magnesium—provided by dairy foods.

Although studies have generally failed to demonstrate that dairy calcium is better absorbed than calcium from mineral salts, the availability for bone mineralisation appears to be greater for dairy foods and the effects may be longer lasting [11]. Consequently, head-to-head intervention trials show bone-health benefits for dairy foods over calcium supplements. In postmenopausal women, those randomised to dairy (low-fat milk and yogurt products, fortified with concentrated milk protein) had greater improvements in arm, pelvis, total spine and total-body bone mineral density (BMD) than those receiving calcium supplements in one trial [26, 27], though not at other sites in another trial [28]. In another randomised trial, cheese was more beneficial than calcium supplements for tibia cortical bone mass accrual in children [22]. The authors suggested that this may be a result of better absorption of dairy calcium, differences in the distribution of intake over the day (small regular amounts of dairy versus two large doses from the supplement) and differences in protein or micronutrient intakes [22]. Greater increases in insulin-like growth factor-I (IGF-I), which favours bone formation, have also been reported for dairy compared with calcium supplements [26].

Dairy sources of calcium are considered to be at least as effective for bone health as calcium supplements, and probably more so [11]. A consensus statement from the Belgian Bone Club suggests that for the non-pharmacological management of osteoporosis, single-nutrient supplements will frequently be inadequate and preference should go to the use of complete supplements or complete foods such as dairy products [29]. Calcium supplementation should only be targeted to those who do not get sufficient calcium from their diet and who are at high risk for osteoporosis and/or fracture [30].

## Bone Health: Effect of Dairy Products Consumption

Bone mineral mass is determined by the amount of bone accumulated at the end of skeletal growth (peak bone mass) and by the amount of bone lost subsequently. Building a strong skeleton from foetal life to adulthood and maintaining healthy bones through the menopause and ageing are vital to minimize frailty in the elderly. After the age of 20 years, there is little change in bone mineral mass until the menopause in women, when a rapid drop in oestrogen leads to an increase in bone remodelling. Menopause is associated with an average annual bone loss of 3–5 % during the first few years and around 1 % thereafter [31]. In men, bone loss is slower and more linear, but men also develop osteoporosis with ageing. The risk of osteoporosis significantly increases after 50 years of age.

There is growing evidence that the consequences of age-related or postmenopausal bone loss on fracture risk depend on the level of peak bone mass achieved during childhood and adolescence, as well as on the rate of bone loss [32]. Maximizing peak bone mass may be an important contributor to fracture risk reduction in children as well as in the elderly. While 60–80 % of the variance in peak bone mass is explained by genetic factors, the remainder may be amenable to interventions aimed at maximizing it [32, 33]. Such interventions include increasing physical activity and decreasing exposure to risk factors such as cigarette smoking and excessive alcohol intake, as well as optimizing nutrition [21, 32]. Adequate dietary calcium and protein intakes are essential to achieve optimal peak bone mass during skeletal growth and to prevent bone loss in the elderly [34].

### Dairy Consumption for Bone Development in Children

The importance of dietary calcium for bone growth is evident even prior to birth. Diets rich in calcium and other micronutrients (as supplied by dairy foods, green leafy vegetables, fruits) given to pregnant women are associated with increased skeletal growth and/or bone mass/density in the offspring, with beneficial effects on bone size and BMD apparent up to the ages of 6–9 years [35, 36]. This is compatible with the hypothesis that dairy consumption by pregnant women might promote bone health in the child.

During childhood, the available data indicate that dairy products are important for growth and bone health [37]. In growing children, long-term milk avoidance is associated with smaller stature, lower bone mineral mass, and increased fracture risk before puberty of around 2.7-fold higher than a matched birth cohort [38, 39]. The largest randomised controlled trial (RCT) with dairy products found significantly higher gains in height, body weight, bone mineral content (BMC) and BMD in school girls aged 10 years receiving milk on school days for 2 years compared with the control group [40]. The beneficial effects of calcium and dairy products on bone mineral mass during growth in children are supported by meta-analyses of numerous clinical studies on milk-derived calcium phosphate supplementation and increased dietary dairy products, with a statistically and clinically higher gain of bone mineral content in those with low basal calcium intake [41, 42]. This significant increase in bone mass following calcium enrichment of the diet observed in pre-pubertal girls and boys [43, 44] was maintained for 1–3 years after the end of the trial [44, 45], suggesting a possible optimization of peak bone mass when calcium supply is sufficient. Increasing dairy product consumption in girls aged 10–12 years was found to be more beneficial for cortical bone mass accrual than calcium supplementation in tablet form for the same calcium intake (1000 mg/d) [22]. Besides mineral intake, a higher protein intake interacts with physical activity to enhance bone mass acquisition before the onset of puberty [3].

In summary, numerous observational studies and RCTs have shown a favourable effect of dairy products on bone health during childhood and adolescence. Conversely, avoiding milk is linked to a lower BMC [22] and increased fracture risk [22] in children.

### Dairy Consumption for Osteoporosis Prevention and Reduction of Fracture Risk in Adults

European guidance on osteoporosis incorporates nutritional recommendations for bone health, including at least 1000 mg/d calcium and 800 IU/d vitamin D [30]. For comparison, Recommended Dietary Allowance (RDA, mg/d) for calcium is 1200 for postmenopausal women according to the US Institute of Medicine [46] and 600 in Japan [47]. Recommended nutritional intakes are 700 in UK [48] and recommended dietary intakes 1300 in Australian postmenopausal women [49]. Although scientific evidence to support the daily dose of calcium is rather weak, recent recommendations for postmenopausal women (aged 50–70 years) include optimal dietary protein intake of 1.0–1.2 g/kg body weight/d with at least 20–25 g of high-quality protein at each main meal, with adequate vitamin D intake at 800 IU/d to maintain serum 25-hydroxyvitamin D levels >50 nmol/L as well as calcium intake of 1000 mg/d, alongside regular physical activity/exercise 3–5 times/week combined with protein intake in close proximity to exercise [50]. Several meta-analyses support the role of calcium and vitamin D (mostly from supplements) for the prevention of osteoporosis [51–53]. As a rich source of calcium, vitamin D and protein, dairy products could be of help for reducing the risk of osteoporosis.

The healthcare costs of osteoporosis are substantial. In a review on the effects of dairy products in several medical conditions, it was suggested that intake of the recommended quantities of dairy products in the USA would yield 5-year savings (limited to healthcare costs) of $209 billion [54]. Of this, $14 billion relate to savings on the healthcare costs for osteoporosis (limited to treating fractures) [54]. A recent study shows that, especially for France, the Netherlands and Sweden, the societal burden of hip fractures associated with low calcium intake is quite substantial. Costs of fractures that might potentially be reduced by improving dairy consumption have been calculated at around €129 million in France and €34 million in Sweden [55].

Protein malnutrition is frequent in the elderly and contributes to the development of osteoporosis. Approximately 2 % (1–8 %) of the variance in BMD/BMC may be explained by dietary protein intake, possibly via an increase in serum IGF-I levels, with a reduction of proximal femur BMD loss and reduction of bone fracture risk [56–59]. However, the role of dietary protein in osteoporosis remains controversial, as diets high in animal protein (and sodium) have been linked to increased urinary calcium excretion [60, 61], which could, in theory, lead to bone loss and osteoporosis [62]. This ‘acid-ash hypothesis’—first proposed 40 years ago—theorizes that high animal protein intake results in metabolic acidosis and increased urinary excretion of calcium. However, recent human studies show that there is no relationship between nutritional variations in urinary acid excretion and calcium balance, bone metabolism and osteoporotic fracture risk [63]. Furthermore, a systematic review and meta-analysis concluded that a causal association between dietary acid load and osteoporotic bone disease is not supported by evidence [64]. The systematic review found weaknesses in the acid-ash hypothesis, in terms of a lack of direct evidence of osteoporosis progression (fragility fractures or bone strength) from the intervention studies, a lack of control for osteoporosis risk factors (such as weight loss, family history of osteoporosis, baseline BMD and oestrogen status) in the supporting prospective cohort studies, no biological mechanism functioning at physiological pH and no evidence for an adverse role on osteoporosis of phosphate, milk and grain foods in the randomised studies [64]. Changes in urinary calcium are not reflected in calcium balance [64], with high protein intakes increasing calcium excretion—but without impairing calcium balance—and possibly increasing intestinal calcium absorption [65]. Intakes of aromatic amino acids, which are prevalent in dairy foods, increase IGF-I and stimulate the intestinal absorption of calcium. A high dairy-protein diet has been demonstrated to increase urinary calcium excretion but at the same time improve calcium intake and attenuate bone loss in overweight individuals on a weight loss and weight maintenance diet [66]. A 2-year controlled trial has shown beneficial effects on BMD of daily alkali supplements [67]. In summary, theories proposing harmful effects of dairies through the production of acid are not supported by evidence.

### Fracture Risk

Although, ideally, clinical outcome measures (fracture incidence) would be used to assess the impact of dairy on osteoporosis, in practice, research studies mostly utilise surrogate outcomes, including BMD [68] and bone turnover markers. Controlled trials show a statistically significant positive association between dairy food intake and BMC or BMD, and an inverse association with bone turnover markers [26, 27].

In terms of actual fracture rates, meta-analyses support some role of calcium and vitamin D (mostly from supplements) for reducing the risk [51–53]. However, globally, the relationship between dietary calcium intakes and fracture rates is not straightforward [69, 70]. Hip fracture rates are in fact highest in countries with high calcium intake and lowest where calcium intake is lower (the opposite to what might be expected) [69]. Caucasian women living in temperate climates have the highest rate of hip fracture, while rates are somewhat lower in Mediterranean and Asian women and lowest in African women [69]. This incongruence may be due to the influence of vitamin D status (linked to latitude) and high levels of bone-protective physical activity in poorer countries with low dairy intakes [71] as well as differences in life expectancy. Furthermore, there is a considerable physiological capacity to adapt to low dietary calcium intakes without compromising bone health [72].

Data on the relationship between dairy food intake and fracture risk are currently limited. The available prospective studies are summarised in Table 3, comprising observational data (albeit many with sizable populations) but no RCTs. Overall, the studies to date have found variable associations between dairy consumption and fracture risk (Table 3).

**Table 3:** Prospective data examining the link between dairy consumption and fracture risk

Study population	Study findings	Reference
Meta-analysis	Meta-analysis of 6 prospective cohort studies (*n* = 39,563 men and women), with 152,000 person-years of follow-up, found that low dairy intake (less than 1 glass of milk daily) was not associated with a significantly increased risk of fracture (any fracture, osteoporotic fracture or hip fracture)	[139]
Meta-analysis	Meta-analysis of 7 prospective cohort studies (195,102 women and 75,149 men, middle aged or older) found that in women there was no association between total milk intake and hip fracture risk, whereas men had a 9 % reduction in relative risk of fracture per daily glass of milk	[73]
Health Professionals Follow-up Study	Prospective cohort study of 43,063 men (40–75 years of age at baseline) with 8-year follow-up concluded that there was no relation between dairy calcium intake and forearm fracture. There was a non-significant trend to reduction of hip fractures with the highest dairy calcium intakes	[140]
Study of Osteoporotic Fractures (SOF Study)	Prospective cohort study of 9704 women (aged ≥ 65 years) with mean 6.6-year follow-up concluded that there was no association between milk intake and the risk of any of the fractures studied. The exception was ankle fractures, which significantly decreased with increasing milk intake	[141]
National Health Screening, Norway	Prospective cohort study of 19,752 women and 20,035 men (middle aged) with mean 11.4-year follow-up found an increased risk of hip fracture for people with a diet that was high in (non-dairy animal) protein and low in milk intake (up to 1 glass of milk per day)	[142]
Japanese Adult Health Study	Prospective cohort study of 4573 people (mean age 58.5 years) found that low milk intake was marginally associated with an increased risk of hip fracture	[143]
Swedish Mammography Cohort	Prospective cohort study of 60,689 women (aged 40–74 years at baseline) found that there was no dose–response relationship between dietary calcium and risk of osteoporotic fracture	[77]
Swedish Mammography cohort and Cohort of Swedish Men	Analysis of two prospective cohort studies, comprising 61,433 women and 45,339 men (aged 39–74 years at baseline), with mean follow-up of 20.1 years found that higher mortality (men and women) and increased fracture (women) were associated with high milk intake. However, high intake of cheese or fermented milk products was associated with lower mortality and fracture rates in women	[78]
Framingham Offspring	Prospective cohort of 3212 men and women (aged 26–85 at baseline) with 12 years of follow-up found a weak protective trend of yogurt (but not other dairies) on risk of hip fracture	[75]
Framingham Original Cohort	Prospective cohort study of 830 men and women with mean 11.6-year follow-up concluded that greater intakes of milk and milk + yogurt may lower risk for hip fracture in older adults	[76]
Nurses’ Health Study	Prospective cohort study of 77,761 women (aged 34–59 years at baseline) with 12 years of follow-up concluded that higher milk consumption did not protect against fracture (hip or forearm)	[74]
Nurses’ Health Study	Prospective cohort study of 72,337 postmenopausal women with 18 years of follow-up found that that milk did not reduce hip fracture risk	[2]
Nurses’ Health Study and Health Professionals Follow-up Study	Prospective cohort study of 35,349 men (aged 40–75 years at baseline) and 61,578 women (30–55 years of age at baseline) with 22-year follow-up found that milk consumption as a teenager was not associated with hip fractures in adulthood	[144]
French Three-City Study	Prospective cohort study of 1482 individuals (aged ≥67 years) with an 8-year follow-up found that low intake of dairy products was associated with an increased risk of wrist fractures	[145]
European Prospective Investigation into Cancer and nutrition study (EPIC)	Prospective study of 10,538 men and 18,584 women (aged ≥60 years), followed up for a median of 8 years, found that dairy consumption did not influence the risk of hip fracture	[146]
Spanish elderly cohort	Prospective cohort study of 5201 women (aged ≥65 years) with 3-year follow-up found that dairy calcium intake less than 250 mg/day was associated with the risk of non-spinal fracture	[147]
European Prospective Osteoporosis Study (EPOS)	Prospective study of 3173 men and 3402 women (aged 50–79 years) with a mean 3.8-year follow-up found that vertebral fracture was not associated with milk consumption	[148]
NHANES I Epidemiologic Follow-Up Study cohort	Prospective cohort study of 4342 men and postmenopausal women (aged 50–74 at baseline), with up to 16 years of follow-up, concluded that dietary calcium may reduce the risk of hip fracture in late menopausal women	[149]

A meta-analysis of prospective cohort studies in middle-aged or older men and women demonstrated no overall association between total milk intake and hip fracture risk in women, with a reduction in relative risk of fracture of 9 % (relative risk 0.91, 95 % CI 0.81–1.01) per daily glass of milk (containing approx. 300 mg calcium) among men, which requires further validation [73]. However, the data on women were strongly influenced by one study from Sweden; the authors excluded this study from the analysis on the basis of its disproportionate influence, whereupon there was a marginally significant 5 % lower hip fracture risk per glass of milk daily (pooled relative risk 0.95, 95 % CI 0.90–1.00, *P* = 0.049) [73].

One of the largest populations with the longest follow-up was the Nurses’ Health Study (Table 3). With a 12-year follow-up, over 77,000 women aged 34–59 years assessed dietary intake of calcium with a food-frequency questionnaire at intervals of 2–4 years, and there was no evidence that higher intakes of milk or calcium from food sources reduce fracture incidence, with relative risks of 1.45 (95 % CI 0.87–2.43) for hip fracture and 1.05 (95 % CI 0.88–1.25) for forearm fracture for women drinking two or more glasses of milk per day compared with women consuming one glass or less per week [74]. Furthermore, women consuming greater amounts of calcium from dairy foods had a modest but significantly increased risk of hip fracture. However, as the authors point out, there was no reason to suggest that dairy calcium itself was responsible for this risk [74]. At 18 years of follow-up, there was similarly no relationship between milk and fracture risk in women [2].

The association between dairy product consumption and hip fracture risk was also examined in a 12-year follow-up of the Framingham Offspring cohort [75]. There was a trend to a correlation between higher milk intake and reduced hip fracture. Participants in the higher tertiles (T2–3) of milk intake had a lower but non-significant risk of hip fracture than those in the lowest tertile (T1) (T2 HR 0.78, 95 % CI 0.37–1.63; T3 HR 0.50, 95 % CI 0.22–1.13, *P* trend = 0.09). Similarly, participants in the higher categories (C2–3) of yogurt intake showed a protective but non-significant association with the risk of hip fracture as compared to participants in the lowest category (C1) (C2 HR 0.39, 95 % CI 0.15–1.02, C3 HR 0.57, 95 % CI 0.19–1.68, *P* trend = 0.10). Participants in the highest tertile (T3) of fluid dairy intake had a lower risk of hip fracture than those in the lowest tertile (T1) (T3 vs T1, *P* = 0.05) (T2 HR 0.92, 95 % CI 0.46–1.87, T3 HR 0.40, 95 % CI 0.17–0.99, *P* trend = 0.06) [75]. A protective effect of milk was also identified in the Framingham Original cohort [76]. When milk intake was analysed as low versus medium/high intake, there was a trend to correlation, with participants with medium (>1 and <7 servings/wk) or higher (≥7 servings/wk) milk intake tending to have lower hip fracture risk than those with low (≤1 servings/wk) intake (high vs low intake: HR 0.58, 95 % CI 0.31–1.06, *P* = 0.078; medium vs low intake: HR 0.61, 95 % CI 0.36–1.08, *P* = 0.071; *P* trend: 0.178) [76]. Participants with medium/high milk intake (>1 serving/wk) had a 40 % lower risk of hip fracture, compared with those with low milk intake (≤1 serving/wk) (medium/high milk intake: HR 0.60, 95 % CI 0.36–1.02, *P* = 0.061) [76]. However, both of these populations [75, 76] were relatively small compared with the largest studies in Table 3.

A prospective study of 60,000 Swedish women aged 40–74 years who completed a self-administered food-frequency questionnaire at baseline found no correlation between dietary calcium or vitamin D intake for the primary prevention of osteoporotic fractures over 11 years of follow-up [77]. However, with 20.1 years of prospective follow-up (61,000 Swedish women aged 39–74), fermented milk products (yogurt and other soured milk products) and cheese consumption were associated with a significant decrease in fracture incidence and with lower mortality [78]. For each serving, the rate of mortality and hip fractures was reduced by 10–15 % (*P* < 0.001). However, in the same study, high intakes of milk (3 or more glasses of milk; more than 600 ml/day) were associated with increased fracture rate, and there was an association between higher cardiovascular mortality and high milk intake at levels exceeding the recommended doses (≥3 glasses/d; ≥600 g/d), along with a lesser effect of milk intake on cancer mortality [78]. This level of consumption is much higher than the mean milk intake of adults of most countries, and it is worth noting that dietary questionnaires were performed in 1987–1990 and 1997, when milk in Sweden was fortified with high dose of vitamin A; high levels of vitamin A intake have been linked to an increased risk of fracture [79].

It comes as no surprise that the findings of studies in this area are variable (Table 3). The studies are observational in nature and vary widely in terms of the age of the participants, background diets and length of follow-up. There is a lack of RCTs investigating the relationship between dairy food intake and fracture risk, which requires further study. Incidence of fracture is the key clinical outcome that needs to be measured in a longitudinal study; however, it is difficult to construct a dairy-free diet for the comparator group that meets nutritional requirements for calcium and vitamin D levels. Dietary studies are always difficult to control, since increasing intake of one nutrient (or foodstuff) may have an impact on other nutrients (or foodstuffs), affecting the overall dietary composition and nutrient density. With multiple nutrients known to affect bone health, it is particularly important that intake of each is considered. Quantitating intake may also be particularly difficult and variable between studies, due to differences in serving size (for example ‘a glass of milk’, ‘a pot of yogurt’). Furthermore, the study of such a long-term effect requires either prohibitively expensive long-term prospective studies or the reliance on the memory of elderly subjects concerning dairy product consumption for periods extending as far back as childhood. Since a wide range of genetic and lifestyle factors affect bone health, there is great population variability and plenty of potentially confounding factors that require controls. Although epidemiological studies potentially allow large sample numbers and stratification for confounding factors, this design does not allow any causal relationship to be established between dairy consumption and fracture risk.

## Other Potential Effects of Dairy Consumption

In this section, some of the perceived beliefs associated with dairy consumption are briefly discussed, including obesity, lactose intolerance, arthritis and cardiovascular disease.

### Dairy Products and Body Weight

Unfavourable body weight is a risk factor for many diseases, including osteoporosis (under weight) and osteoarthritis (over weight) [80], and obesity is a concern of many patients and health professionals alike. Dairy consumption has been studied extensively for its possible roles in body weight regulation. However, the data are conflicting. Four reviews or meta-analyses concluded that neither calcium nor dairy intakes were reliably demonstrated to aid in weight loss with or without caloric restriction [81–84]. There is evidence to suggest that consumption of dairy products reduces body fat but not necessarily body weight, due to a preservation of lean body mass [85–87]; and recent evidence from prospective cohort studies suggests a protective effect of dairy consumption on risk of overweight and obesity [88, 89]. Consumption of dairy products has been associated with decreased prevalence of metabolic-related disorders [90], and even high-fat dairy consumption within typical dietary patterns has been inversely associated with risk of obesity.

The effect of dairy consumption on weight and body composition has been investigated in two recent meta-analyses [86, 87]. The first meta-analysis of 14 RCTs involving 883 adults found that increasing dairy consumption to recommended daily intake levels in adults who do not follow any calorie-restricted diet had a small effect on weight loss, but also a decrease in fat mass and waist circumference and an increase in lean body mass [86]. When combined with energy restriction, dairy intake showed a modest additional benefit on weight reduction (−0.61 kg, 95 % CI −1.29 to 0.07, *P* = 0.08), greater reduction in fat mass (−0.72 kg, 95 % CI −1.29 to −0.14, *P* = 0.01), gain in lean mass (+0.58 kg, 95 % CI 0.18–0.99, *P* < 0.01) and reduction in waist circumference (−2.19 cm, 95 % CI −3.42 to −0.96, *P* < 0.001) compared with controls [86]. The second meta-analysis of 29 RCTs involving 2101 participants found that overall consumption of dairy products did not result in a significant reduction in weight. There was no significant difference in body weight changes between the dairy intervention and control groups (−0.14 kg, 95 % CI −0.66 to 0.38 kg); however, a significant reduction that favoured dairy products in body fat was shown (−0.45 kg, 95 % CI −0.79 to –0.11 kg) [87]. Dairy products significantly reduced body weight in the short-term interventions (−0.47 kg, 95 % CI −0.90 to −0.03 kg) but moderately increased weight gain in long-term interventions (+0.66 kg, 95 % CI −0.14 to 1.46 kg). However, in the context of energy restriction, the sub-group analysis demonstrated that consumption of dairy products reduced body weight [87]. These two meta-analyses suggest that dairy intake can aid weight and body fat loss.

Other studies show the beneficial impact of specific dairy products on weight management, particularly yogurt. Changes in diet and lifestyle factors were evaluated prospectively to determine their impact on long-term weight gain among 22,557 men and 98,320 women included in health studies in the USA [85]. Over a 4-year period, it was found that yogurt consumption was associated with a significant reduction of weight gain [85]. In another study, a cohort of 8516 Mediterranean men and women was prospectively evaluated [91]. A high (≥7 servings/wk) consumption of total and whole-fat yogurt was associated with lower incidence of overweight/obesity in comparison with low consumption (0–2 servings/wk) [91]. Together, these studies suggest a benefit of including yogurt in the diet in terms of weight management.

Mechanistic explanations for the possible association between high dairy intake and lower body weight/body fat mass found in observational studies include an effect of increased dairy calcium intake on energy balance [92, 93]. Epidemiological data have shown that low calcium intake is a risk factor for overweight and obesity. The clinical implications of this relationship have been confirmed in weight loss studies performed in low-calcium consumers in whom calcium or dairy supplementation accentuated body weight and fat loss [94–96]. Studies have demonstrated that this effect may be explained by an increase in fat oxidation and fat faecal loss as well as a facilitation of appetite control. Indeed, dietary calcium may interfere with fat absorption in the intestine by forming insoluble calcium soaps with fatty acids and/or binding of bile acids, resulting in a decrease in the digestible energy of the diet.

Dairy foods may also modulate body weight regulation by calcium independent mechanisms. Dairy proteins suppress short-term food intake, increase satiety and stimulate food intake regulatory mechanisms known to signal satiation and satiety. Milk proteins may be another important factor explaining the association between dairy consumption and healthier body weights [97].

### Lactose Intolerance

In some people, milk ingestion causes symptoms of bloating, abdominal pain, flatulence and diarrhoea that can be severe enough to prompt avoidance of all dairy foods. The symptoms are caused by a deficiency of the enzyme lactase (the lactose-digesting enzyme that breaks down lactose into galactose and glucose for absorption) causing undigested lactose to increase the osmolarity in the small intestine (causing diarrhoea) and enter the colon where it is fermented by the resident microflora, resulting in gastrointestinal symptoms. While self-diagnosis of lactase deficiency is common, it is often incorrect and leads to unnecessary elimination of dairy products and their nutritional components. It may also result in osteoporosis, since an increased incidence of lactose intolerance (along with a significantly lower daily intake of calcium derived from milk) has been found among women with ‘idiopathic’ osteoporosis [98, 99].

Some years ago, the only possible treatment for lactose intolerance was the avoidance of lactose-containing foods, including most dairy products. Indeed, many believe that lactose intolerant individuals should not consume dairy products. However, the avoidance of dairy products in people with lactose intolerance is an area of controversy. Most individuals with lactose intolerance can tolerate up to 12 g of lactose (250 ml of milk, representing 300 mg of calcium and 30 % of recommended calcium intakes) without suffering gastrointestinal symptoms, although symptoms become more prominent at doses above 12 g and are appreciable after 24 g of lactose [100, 101]. The 2010 Dietary Guidelines for Americans encourage daily consumption of dairy foods and concur that it is important for people with lactose intolerance to obtain the health and nutritional benefits associated with dairy products, recommending yogurt or cheese, or smaller portions, and lactose-free varieties where necessary [13]. A National Institutes of Health (NIH) Consensus and State-of-the-Science Statement confirms that even in persons with lactose maldigestion, small amounts of milk, yogurt and hard cheese, particularly if ingested with other foods and distributed throughout the day, and reduced-lactose food may be effective management approaches, though the amount of lactose subjects with lactose intolerance can take, is based on low-quality evidence [102].

Yogurts (those with the two active bacterial cultures: *Lactobacillus delbrueckii* subsp. *bulgaricus* and *Streptococcus thermophilus*) and hard cheeses contain more pre-digested lactose and may be more readily tolerated than fluid milk [103, 104]. The bacterial lactase survives the acidic conditions of the stomach, apparently being physically protected within the bacterial cells and facilitated by the buffering capacity of yogurt. The increasing pH, as the yogurt enters the small intestine, and a slower gastrointestinal transit time allow the bacterial lactase to be active, digesting lactose from yogurt sufficiently to prevent symptoms in lactose intolerant people [104]. Consequently, the avoidance of all dairy products in lactose intolerant patients is no longer recommended. Dietetic advice should aim to ensure nutritional adequacy of the diet and to avoid nutritional deficiencies—in particular a low calcium intake. Since yogurt and hard cheese in particular are easily digestible and well tolerated by people who find lactose difficult to digest, they can be recommended as part of a balanced diet to help lactose intolerant people take advantage of the nutritional benefits of dairy products [105].

In summary, to varying degrees depending on the cultural history of dairy consumption, genetic lactase persistence allows most of the population to continue to consume some milk beyond the weaning period without gastrointestinal adverse effects. For people who are lactose intolerant, it is no longer necessary to avoid all dairy foods, and in particular yogurt or hard cheese are well tolerated and provide the nutritional benefits of dairy products.

### Dairy Products and Joint Diseases

In people with chronic diseases, dietary manipulation is common as they try to alleviate the symptoms. This is particularly the case with painful conditions such as osteoarthritis (OA) and rheumatoid arthritis (RA). However, scientific studies are required to control for all the possible confounding factors and bias that might contribute to any perceived dietary effect, and the available evidence suggests that there is no reason why dairy consumption should be avoided.

Best practice guidelines for OA emphasize self-management including weight control and exercise [106]. There is some evidence to suggest that a Western diet and inactivity are pro-inflammatory and that low-grade inflammation and oxidative stress underlying OA often coexist with lifestyle-related risk factors and conditions [107]. While dairy products have in the past been considered as pro-inflammatory [107], more recent data do not support this hypothesis. In fact, full-fat dairy products and dairy fats have either a neutral or inverse effect on levels of circulating inflammatory biomarkers [108–112]. A meta-analysis of nutritional interventional studies performed in overweight or obese people found that dairy product consumption does not exert adverse effects on inflammatory biomarkers [113], and in an RCT among subjects with low-grade systemic inflammation, consumption of a combination of low-fat and high-fat dairy products as part of a healthy diet had no adverse effects on inflammation [114].

An association between frequent milk consumption and reduced OA progression has been found in a recent study in women, although no significant association was found in men. Analysis of 2148 participants in the Osteoarthritis Initiative identified a significant dose–response relationship between baseline milk intake and OA progression in women over a 4-year follow-up period [115]. Replication of these novel findings in other prospective studies demonstrating that increase in milk consumption leads to delay in knee OA progression is needed.

For decades, patients have used different diets to try to improve the symptoms of RA, and dietary manipulation is still widely used today. A wide range of conflicting dietary advice is available, yet a lack of scientific information regarding its efficacy often leaves sufferers vulnerable. The common dietary regimens used by people with RA include vegetarian or vegan, Mediterranean, ‘elemental’ and ‘elimination’ diets (including dairy-free diets).

Diet, nutrition and weight loss have shown some promise in alleviating some of the RA disease burden [116]. Prospective studies suggest that dietary antioxidants may be protective for RA, while high coffee consumption, alcohol intake, smoking and obesity increase the risk of developing RA [117]. Some studies suggest that certain dietary elements including polyunsaturated fatty acids (PUFAs), the Mediterranean diet and antioxidants have anti-inflammatory effects and decrease RA disease activity [118, 119].

However, the effects of dietary manipulation on RA are still uncertain due to the studies being small, single trials with moderate to high risk of bias [120, 121]. A Cochrane review of 14 RCTs and 1 controlled clinical trial, with a total of 837 patients, assessed the potential benefits and harms associated with certain dietary regimens in RA [121]. Due to heterogeneity of interventions and outcomes, baseline imbalance and inadequate data reporting, no overall effects were detected and the effects of vegan and elimination diets were uncertain [121].

Dairy products contain a high content of saturated fatty acids, and many of the shorter chain fatty acids found in milk fat have beneficial health effects, with important immune response functions. There is also evidence that the proteins, fats and calcium in milk are beneficial in inflammation [122]. Given the lack of understanding of the nutritional requirements in RA, plus the variability in its clinical course, it is difficult to produce specific dietary recommendations for RA. Elimination diets, particularly those with low intakes of dairy products, should be discouraged [123], and suspected food intolerance tested under strict clinical supervision [124]. Nutrient mega-dosing is inadvisable, although dietary supplementation with calcium, vitamin D, folic acid or multivitamins and minerals may be recommended where necessary [124].

In summary, more research is needed, but based on the available data, there appears to be no evidence why patients with OA or RA should avoid dairy consumption, considering the overall beneficial effect of dairy products for health.

### Dairy Consumption and Cardiovascular Disease

Globally, cardiovascular disease is a major cause of mortality [125], and the influence of diet on known cardiovascular risk factors such as blood pressure, serum cholesterol and body weight, is well established. Consequently, the DASH (Dietary Approaches to Stop Hypertension)-style diet—which incorporates moderate amounts of low-fat dairy—protects against cardiovascular disease, coronary heart disease, stroke and heart failure [126].

A review of the evidence concluded that most studies report no association between dairy consumption and increased risk of cardiovascular disease [127], with a meta-analysis of RCT data finding no significant impact on various cardio-metabolic risk factors following increased dairy consumption (mean increase of 3.6 serves per day), aside from a slight increase in body weight [128]. In fact, dairy consumption may be protective against cardiovascular disease, with beneficial effects demonstrated on known risk factors such as hypertension [129, 130] as well as clinical outcomes [131–133]. For example, meta-analyses of observational data have reported inverse associations between overall cardiovascular disease and milk intake (4 studies; RR 0.94 per 200 mL/d, 95 % CI 0.89–0.99) [132] and dairy consumption (9 studies; RR 0.88, 95 % CI 0.81–0.96) [131]. There was also an inverse association between dairy consumption and overall risk of stroke (12 studies; RR 0.87, 95 % CI 0.77–0.99) [131], whilst another meta-analysis found that total dairy (RR 0.88, 95 % CI 0.82–0.94), low-fat dairy (RR 0.91, 95 % CI 0.85 to 0.97), fermented milk (RR 0.80, 95 % CI 0.71–0.89) and cheese (RR 0.94, 95 % CI 0.89–0.995)] were significantly associated with reduced risk of stroke [133]. There was no significant association between the risk of stroke and intake of full-fat dairy, non-fermented milk, butter and cream [133].

There is currently a lack of research into the effect of full-fat dairy on cardiovascular disease [127], and the relative impact of high-fat dairy versus low-fat dairy is difficult to establish from the current evidence base. European and American guidelines for preventing cardiovascular disease recommend a reduction in total dietary saturated fat [134, 135]. However, it is becoming clear that all saturated fatty acids are not equal. For example, in a study using a food-frequency questionnaire, a higher intake of saturated fatty acids from meat increased incident cardiovascular disease, whereas higher intake of saturated fatty acids from dairy reduced the risk [136]. Research into understanding the metabolic effects of individual fatty acids is still in the early stages, but it would seem that structural characteristics of the individual fatty acid and the food matrix within which it is consumed are likely to have an effect. A meta-analysis of prospective cohort studies reporting circulating fatty acid composition revealed the variable effects of individual saturated fatty acids, with a higher coronary risk for palmitic (RR 1.15, 95 % CI 0.96–1.37) and stearic (RR 1.23, 95 % CI 0.93–1.61) acids, and a significantly lower risk for margaric acid (RR 0.77, 95 % CI 0.63–0.93) [137]. Whilst the saturated fatty acids in whole milk and butter increase low-density lipoprotein, they also increase high-density lipoprotein, which leaves the ratio of total cholesterol to high-density lipoprotein unchanged, or slightly lowered [127].

A recent meta-analysis has concluded that calcium supplements without co-administered vitamin D were associated with an increased risk of myocardial infarction [138]. However, there was no increased risk when calcium was of dietary origin.

In summary, dairy consumption does not seem to significantly increase the risk of cardiovascular disease, particularly if low-fat, and more research is needed for full-fat dairy products.

### Discussion

Milk provides a package of essential nutrients that are difficult to obtain in low-dairy or dairy-free diets. Dairy products may represent a useful source of dietary calcium, and for many people it is not possible to achieve recommended daily calcium intakes with a dairy-free diet. The link between dairy product intakes and fracture risk reduction (Table 3) is still challenged. To demonstrate an effect on fracture risk would require further large and well-designed rigorously controlled studies.

Nevertheless, it can be inferred from the above-mentioned data that regular consumption of dairy throughout life is likely to be beneficial for skeletal health. Besides bone health, more controversial are the beliefs around including dairy as part of a healthy balanced diet. Among some members of the public and health professionals, doubts persist that dairy may be detrimental to health. In some cases, these are simply beliefs or a consequence of misinformation, but in other cases plausible mechanisms exist, casting doubt in even the most scientific mind. This review has discussed some of the most common concerns over the inclusion of dairy in the diet, including those of people with weight management issues or lactose intolerance, with OA or RA or with cardiovascular disease. The overall message is that the benefits outweigh any (in many cases perceived, not real) harm.

Lactose intolerance can prompt an avoidance of all dairies, but this is not necessary in most people. In particular, yogurt and hard cheese are well tolerated and provide the nutritional benefits of dairy products.

The effect of dairy consumption on body weight and composition has been investigated extensively, with conflicting results. There may be a weak association between dairy consumption and a possible small weight reduction, with decreases in fat mass and waist circumference and increases in lean body mass.

The evidence for any role of dairy foods in inflammation is conflicting and limited to small observational or clinical trials. Although dairy foods have in the past been considered pro-inflammatory [107], studies of dairy consumption demonstrate that full-fat dairy products and dairy fats in fact have either a neutral or inverse effect on levels of circulating inflammatory biomarkers [108–114]. Recently, a single observational study suggested a reduction in OA progression associated with frequent milk consumption among women [115]. Dietary guidelines are difficult to define for RA, but patients should be discouraged from eliminating dairy [123] in their attempts to control their symptoms, and a balanced nutritionally complete diet should be advised.

Most studies report no association between dairy consumption and increased risk of cardiovascular disease [127], and in fact low-fat dairy may be protective [131–133]. More research is needed in this respect for full-fat dairy products.

In conclusion, dairy products may represent a valuable dietary source of calcium due to their high calcium and nutrient contents, high absorptive rate, availability and relatively low cost. Numerous national nutritional recommendations are for 3 servings of dairy products per day (for example, 1 glass of milk, 1 portion of cheese, 1 yogurt), an amount that provides the recommended daily intake of calcium. By understanding fully the accumulating scientific data in this area, health professionals can play an important role in dispelling these beliefs surrounding dairy products.
